# Comparative gene expression profiling of placentas from patients with severe pre-eclampsia and unexplained fetal growth restriction

**DOI:** 10.1186/1477-7827-9-107

**Published:** 2011-08-02

**Authors:** Haruki Nishizawa, Sayuri Ota, Machiko Suzuki, Takema Kato, Takao Sekiya, Hiroki Kurahashi, Yasuhiro Udagawa

**Affiliations:** 1 Department of Obstetrics and Gynecology, Fujita Health University School of Medicine, Fujita Health University, Toyoake, Japan; 2 Division of Molecular Genetics, Institute for Comprehensive Medical Science, Fujita Health University, Toyoake, Japan

## Abstract

**Background:**

It has been well documented that pre-eclampsia and unexplained fetal growth restriction (FGR) have a common etiological background, but little is known about their linkage at the molecular level. The aim of this study was to further investigate the mechanisms underlying pre-eclampsia and unexplained FGR.

**Methods:**

We analyzed differentially expressed genes in placental tissue from severe pre-eclamptic pregnancies (*n *= 8) and normotensive pregnancies with or (*n *= 8) without FGR (*n *= 8) using a microarray method.

**Results:**

A subset of the FGR samples showed a high correlation coefficient overall in the microarray data from the pre-eclampsia samples. Many genes that are known to be up-regulated in pre-eclampsia are also up-regulated in FGR, including the anti-angiogenic factors, *FLT1 *and *ENG*, believed to be associated with the onset of maternal symptoms of pre-eclampsia. A total of 62 genes were found to be differentially expressed in both disorders. However, gene set enrichment analysis for these differentially expressed genes further revealed higher expression of TP53-downstream genes in pre-eclampsia compared with FGR. TP53-downstream apoptosis-related genes, such as *BCL6 *and *BAX*, were found to be significantly more up-regulated in pre-eclampsia than in FGR, although the caspases are expressed at equivalent levels.

**Conclusions:**

Our current data indicate a common pathophysiology for FGR and pre-eclampsia, leading to an up-regulation of placental anti-angiogenic factors. However, our findings also suggest that it may possibly be the excretion of these factors into the maternal circulation through the TP53-mediated early-stage apoptosis of trophoblasts that leads to the maternal symptoms of pre-eclampsia.

## Background

Pre-eclampsia is one of the most common and potentially serious pregnancy-associated disorders and is a principal cause of maternal morbidity, accounting for almost 15-20% of pregnancy-related mortalities [1]. Pre-eclampsia is not a simple complication of pregnancy, but is a syndrome involving multiple organ failure including that of the liver, kidney, lung, and the coagulatory and neural systems. The prognosis for both the mother and fetus in cases of severe pre-eclampsia is poorer than generally expected, particularly in early onset cases (<34 weeks of gestation) [2]. Although these patients are generally treated for prevention of seizures and control of hypertension, the lack of understanding of the precise etiology of pre-eclampsia has hindered the development of preventive and therapeutic measures to treat this disease based on its etiology. Since the risks of maternal multi-organ dysfunction and fetal distress are higher in the early onset forms of this disorder, it has been recommended that the fetus should be delivered during gestational weeks 32-34 in such cases [1].

There is now an emerging consensus that pre-eclampsia is a complex polygenetic trait in which maternal and fetal genes, as well as environmental factors, are involved. However, the pathogenetic process involves numerous factors such as oxidative stress, endothelial dysfunction, vasoconstriction, metabolic changes, thrombotic disorders and inflammatory responses, and the precise underlying mechanisms have remained elusive [3,4]. It has also been formally considered, however, that the placenta plays a primary role in the etiology of this disorder. In support of this idea is the observation that patients with pre-eclampsia recover from clinical symptoms of the disorder just after the delivery of the fetus and placenta, that pre-eclampsia occurs in patients with a hydatidiform mole characterized by trophoblast hyperplasia in the absence of any tissues of fetal origin, and also that paternity is a significant risk factor for pre-eclampsia [5]. Several recent lines of evidence have also indicated that placenta-derived anti-angiogenic factors, such as soluble fms-like tyrosine kinase-1 and soluble endoglin, significantly contribute to the onset of pre-eclampsia [6,7].

Fetal growth restriction (FGR) is another clinical entity that increases the risk of perinatal morbidity and mortality and can result from heterogeneous causes, including maternal, fetal and placental factors. FGR may result from defects in endogenous developmental and growth factors, whereas a placental defect may inhibit the transport of nutrients and oxygen from mother to fetus [8]. Some forms of unexplained FGR have been etiologically linked to pre-eclampsia since they show common pathologic features in the placenta, although this assertion has remained controversial [9]. A failure of trophoblast invasion leading to abnormal shallow vascularity and impaired remodeling of the spiral arteries appears to be a common process behind these disorders [10]. Although pre-eclamptic placentas generally manifest fewer morphological changes than those of FGR cases, the placentas from early-onset cases of pre-eclampsia display substantial villous and vascular abnormalities [11,12]. In this context, it could be hypothesized that a subset of FGR and early-onset pre-eclampsia cases are likely to share common etiologies, and that the presence or absence of maternal risk factors determines disease manifestation, but this contention remains speculative [13].

Given the current evidence base, it is of clinical importance to compare the gene expression profiles of pre-eclampsia and FGR to further our understanding of their respective etiologies and aid in the future development of new therapies. In our current study, we have performed such global gene expression profiling via oligonucleotide microarrays of placental tissue from severe pre-eclampsia, unexplained FGR and normal pregnancies.

## Methods

### Human subjects

All clinical samples were collected at the Department of Obstetrics and Gynecology, Fujita Health University, Japan. Informed consent was obtained from each patient and the study protocol was approved by the Ethical Review Board for Human Genome Studies at Fujita Health University (Accession number 43 and 87, approved on February 23, 2005 and March 24, 2010, respectively). A total of 24 placental biopsies were obtained from severe pre-eclamptic pregnancies (*n *= 8) and normotensive pregnancies with (*n *= 8) or without FGR (*n *= 8). Pre-eclampsia was defined as a blood pressure of higher than 140/90 mmHg, with proteinuria of more than 0.3g in a 24 hour collection. The pre-eclampsia was considered severe if one or more of the following criteria were present: a blood pressure of higher than 160/110 mmHg, or proteinuria of more than 2g in a 24 hour collection [14]. FGR was diagnosed when the birth weight was below the 10th percentile of that anticipated for the given gestational age for a Japanese population [15]. Unexplained FGR was defined by the exclusion of known maternal and fetal factors including maternal systemic diseases, multiple gestation, fetal congenital infection, structural abnormalities and chromosomal abnormalities. Additionally, all FGR cases showed clinical signs of disturbed placental function such as asymmetric growth, oligohydroamnions, and/or increased pulsatility index of the umbilical artery. Normotensive subjects with or without FGR were matched for maternal and gestational ages, and for body mass index during pre-pregnancy (Table 1).

**Table 1:** Clinical parameters of the study groups

	Control	FGR	PE	*P *Value		
	(n = 8)	(n = 8)	(n = 8)	Control vs FGR	Control vs PE	FGR vs PE
Maternal age (y)	31.5 ± 6.5^a^	31.4 ± 3.7	31.0 ± 4.7	0.96	0.86	0.86
Prepregnancy Body mass index	21.4 ± 2.3	19.9 ± 1.9	21.7 ± 3.7	0.19	0.83	0.26
Gestational age at delivery (wks)	38.1 ± 0.8	37.3 ± 1.0	34.4 ± 1.8	0.08	<0.01	<0.01
Birth weight (g)	2891.5 ± 309.6	1765.4 ± 483.9	1666.6 ± 441.0	<0.01	<0.01	0.68
Birth weight coefficient	1.007 ± 0.120	0.646 ± 0.151	0.778 ± 0.103	<0.01	<0.01	0.06
Placental weight (g)	571.4 ± 151.0	329.4 ± 61.3	341.3 ± 59.4	<0.01	<0.01	0.70
Systolic BP (mmHg)	111.3 ± 11.1	123.3 ± 14.5	160.6 ± 10.0	0.08	<0.01	<0.01
Diastolic BP (mmHg)	66.9 ± 8.7	77.0 ± 11.9	105.3 ± 9.6	0.07	<0.01	<0.01
Proteinuria (%)^b^	0	0	100	n.s.	<0.01	<0.01
UmA PI^c^	0.76 ± 0.10	1.11 ± 0.32	1.12 ± 0.34	0.01	0.01	0.98

^a ^Data are given as the mean ± standard deviation (SD)

^b ^≥ 5g in a 24 hour collection

^c ^Pulsatility index of the umbilical artery

### Placental biopsy collection

All of the placental biopsies, both from pre-eclamptic and normotensive pregnancies with or without FGR, were obtained following Caesarean sections. In normotensive cases, Caesarean sections were performed due to previous Caesarean sections. To avoid any effects of labor upon the gene expression profiles of the tissue samples, only placental samples that were obtained from the women who had not undergone labor were included in the study. A central area of chorionic tissue was dissected, and the maternal deciduas and amnionic membranes were removed. We then dissected 1 cm sections of placental villi from the four different central areas between the basal and chorionic plates. After vigorous washing of the maternal blood with saline, tissues were immediately frozen in liquid nitrogen and stored until use.

### RNA extraction

Total RNA was extracted from the chorionic villous tissues with an RNeasy mini-kit (Qiagen Inc., Valencia, CA) in accordance with the manufacturer's instructions. The quality of the RNA samples was determined by electrophoresis through denaturing agarose gels and staining with ethidium bromide. We measured the intensities of 28S and 18S rRNAs and a higher than 2:1 ratio was considered the benchmark for intact RNA. The RNA was quantified and evaluated for purity by UV spectrophotometry. RNA samples obtained from four different areas of a single placenta were mixed prior to expression analyses. To further assess the quality of the RNA, all specimens were subjected to expression analysis of the housekeeping gene, glyceraldehyde-3-phosphate dehydrogenase (GAPDH), using conventional RT-PCR. To test for possible contamination by maternal blood, the expression of a leukocyte-specific gene (leukocyte common antigen; LCA) was also examined using conventional semi-quantitative RT-PCR [16].

### Microarray analysis

Microarray experiments were performed using the Affymetrix GeneChip system (Affymetrix, Santa Clara, CA). Briefly, approximately 1 μg of each total RNA sample was used for double strand cDNA synthesis. cRNA was synthesized by *in vitro *transcription followed by sense-strand cDNA synthesis using the Ambion Whole Transcript Expression Kit (Applied Biosystems, Foster City, CA). After fragmentation into ~200 bp strands with uracil DNA glycosylase, synthesized sense-strand cDNAs were end-labeled with biotin, and then hybridized to an Affymetrix Human Exon 1.0 ST Array. Signal intensities were amplified via secondary staining with a biotin-labeled anti-streptavidin antibody followed by phycoerythrin-streptavidin staining. Fluorescent image arrays were obtained using a GeneChip Scanner 3000 (Affymetrix).

Microarray data analyses were performed using Microsoft Excel, Partek Genomics Suite 6.5 (Partek Inc., St. Louis, MO) and Genespring GX11 (Silicon Genetics, Redwood City, CA). All data were subjected to per chip and per gene normalization, quantile normalization and quality control, and a total of 24,805 genes were used for further analysis. Microarray data were deposited in the GEO database with the detailed hybridization information according to the MIAME guidelines and assigned the accession number GSE24129 [17]. To analyze the correlation of microarray data pairs, we used the Pearson's correlation coefficient. In addition, we adopted the non-negative matrix factorization (NMF) method, which is an unsupervised classification algorithm [18,19]. The NMF method was performed using the NMF package in the R system. *K *= 2-5 indicates the number of subclasses modeled. To identify specific pathways that may be involved in the progression of pre-eclampsia or FGR, we performed standard gene ontology (GO) analysis using the Gene Spring software. We also performed gene set enrichment analysis (GSEA), a computational method that determines whether defined sets of genes show statistically significant, concordant differences between two biological states [20,21]. For this analysis we used GSEA v2.06 software from the Broad Institute [22]. Gene sets were downloaded from the Molecular Signatures Database (MSigDB) of this web site. We used the C2 curated catalogue of functional gene sets to select significant gene sets. A false discovery rate (FDR) of less than 0.25 and *P *values of less than 0.001 were considered significant.

### Real-time RT-PCR

To validate the microarray data for selected genes, we performed quantitative real-time RT-PCR (qRT-PCR) analysis using the Taqman System (Applied Biosystems, Foster City, CA). The Superscript First-strand Synthesis System for RT-PCR (Invitrogen, Carlsbad, CA) with random primers was used to produce single stranded cDNA templates. A housekeeping gene, *ACTB*, was used to normalize for mRNA levels because its expression levels among the samples were stable. All RT-PCR reactions were performed in triplicate in a final volume of 25 μl. The cycling conditions used for PCR amplification were 2 min at 50°C, 30 min at 60°C and 1 min at 95°C for RT, followed by 40 cycles of 15 sec at 95°C and 1 min at 60°C. For qRT-PCR, we increased the number of samples in addition to the original 24 samples such that a total of 41 pre-eclampsia, 16 FGR and 45 normotensive pregnancies were analyzed.

#### Statistical analysis

Statistical comparisons between groups were performed using the Student's *t *test and one-way analysis of variance (ANOVA) and differences were considered to be significant at *P *< 0.05.

## Results

**Figure 1:**
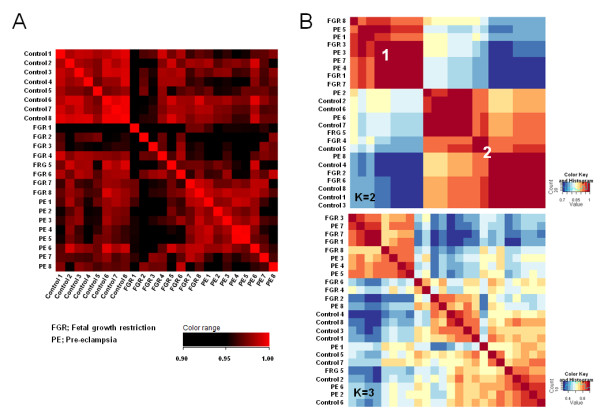
**Overall gene expression profile analysis of the placentas from pre-eclamptic and normotensive pregnancies with or without FGR**. **A**. Correlation coefficient of the microarray data between each sample pair. Red indicates 1.0, whereas black indicates less than 0.9. PE, pre-eclampsia. **B**. Clustering analysis using the NMF method. *K *= 2 (upper panel) or *K *= 3 (lower panel) indicate the number of modeled subclasses. Red indicates a high, whereas blue indicates a low correlation coefficient. The cophenetic correlation coefficients were measured as follows: 0.9313 (*K *= 2), 0.8350 (*K *= 3), 0.7915 (*K *= 4), and 0.7838 (*K *= 5).

We compared the global gene expression profiles of human placentas from pre-eclamptic and unexplained FGR cases using oligonucleotide microarrays. Eight samples were used from each of the patient groups and a normotensive control group. To perform an overview of the expression profiles of these samples, we first analyzed the correlation coefficient for the overall microarray data for each sample pair. Comparisons of the samples from any two normotensive controls showed good concordance and a high correlation coefficient (mean correlation coefficient: 0.980; Figure 1A). In contrast, a lack of concordance was found between the normotensive and disease samples (control versus pre-eclampsia, 0.967; control versus FGR, 0.965), indicating that the expression of numerous genes is altered in the placenta upon the onset of pre-eclampsia and FGR (Figure 1A). In the pre-eclamptic samples, most sample pairing (FGR1-6) also showed a high correlation coefficient (0.976), whereas less correlation was observed between FGR samples (0.956), raising a possibility of heterogeneous pathophysiology for FGR. Notably also, a subset of FGR samples (FGR 7 and FRG8) showed high correlation coefficient with the pre-eclampsia samples (0.977), suggestive of a common etiological background. This might also be attributable to the fact that pre-eclamptic women delivered newborns with lower birth weight percentiles (Table 1).

To further analyze the correlations for each sample pair, we employed the NMF method to classify the samples based on the overall microarray data. Based on the cophenetic correlation coefficient (coph) calculated for each experiment, *K *= 2 showed the highest value (coph = 0.9313), indicating that NMF class assignment for *K *= 2 was the most robust. In addition, whereas all of the normotensive controls were classified as Group 2, most of the pre-eclampsia samples were classified as Group 1. A subset of the FGR samples was also classified as Group 1, suggesting a common pathophysiology with pre-eclampsia for these cases (Figure 1B).

**Figure 2:**
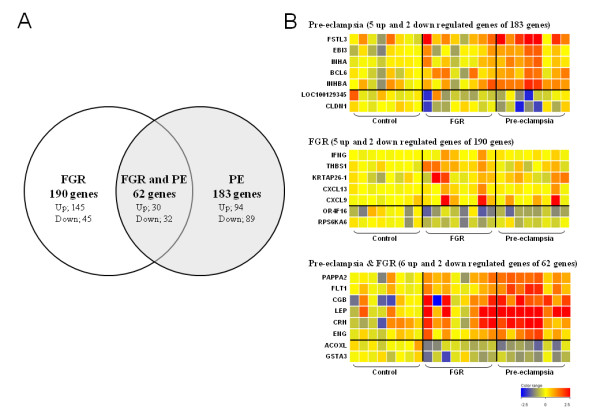
**Comparative gene expression profiling of patients with severe pre-eclampsia, unexplained FGR, and normotensive control pregnancies (n = 8 in each group)**. **A**. Venn diagram of the genes found to be differentially expressed between pre-eclamptic and normotensive control subjects as well as between FGR patients and controls. **B**. The differential expression profile of representative genes. Upper panel, genes differentially expressed in pre-eclampsia; middle panel, FGR; lower panel, in both pre-eclampsia and FGR. Each column represents a single subject and each row indicates a single gene. Gene names are indicated on the left side. The red color indicates up-regulation and blue depicts down-regulation relative to the controls.

To identify differentially expressed genes in a statistically significant manner, we performed ANOVA and fold-change analysis of the data obtained from each sample group. The gene list obtained from a class comparison between the normotensive control and pre-eclamptic pregnancies (*P *< 0.05) was filtered to identify candidates for which the expression levels differed by at least 1.5-fold between the two groups. We thereby identified a total of 245 genes. Similar analysis between the control pregnancy and FGR groups identified a total of 252 genes, including 62 genes in common with the gene set for pre-eclampsia (Figure 2A). As expected, *INHBA*, *INHA *and *FLRG *(*FSTL3*) were found in the pre-eclampsia gene set only, consistent with their previously described association with this disease [23,24]. Our current data were also mainly consistent with our earlier findings [16]. However, many genes that had been reported previously to be upregulated in pre-eclampsia, such as *LEP*, *CGB*, *CRH *and *PAPP-A2 *[25,26], were identified in the gene sets for both pre-eclampsia and unexplained FGR in our present analyses (Tables 2, 3 and 4, Figure 2B, Additional file 1, Table S1). Interestingly, *FLT1 *and *ENG*, which are likely to be associated with the maternal symptoms of pre-eclampsia [6,7], were also found to be significantly up-regulated in both pre-eclampsia and unexplained FGR in our current study. Our present data thus also support the hypothesis that pre-eclampsia and a subset of unexplained FGR share a common background etiology.

**Table 2:** The most highly differentially expressed genes in the pre-eclampsia samples

Gene name	Genbank	Fold-Change	*P *Value
**Up-regulated genes**			
FSTL3	NM_005860	3.30	0.0028
SERPINA3	NM_001085	2.90	0.0137
INHBA	NM_002192	2.42	0.0004
EBI3	NM_005755	2.08	0.0016
TREM1	NM_018643	2.05	0.0062
BCL6	NM_001706	2.02	0.0024
RNU2-1	NR_002716	1.99	0.0013
SLCO2A1	NM_005630	1.91	0.0053
INHA	NM_002191	1.90	0.0019
SNORD115-5	NR_003297	1.87	0.0481
PVRL4	NM_030916	1.80	0.0030
GBA	NM_000157	1.80	0.0022
DERL3	NM_198440	1.78	<0.0001
KRT19	NM_002276	1.75	0.0071
LOC728220	ENST00000391570	1.75	0.0369
**Down-regulated genes**			
CLDN1	NM_021101	2.23	0.0051
LOC100129345	XM_001724751	2.18	0.0165
FAM164A	AF151820	2.15	0.0003
C20orf69	BC118988	2.00	0.0011
ZNF117	NM_015852	2.00	0.0044
ANKRD20B	NR_003366	1.98	0.0230
PART1	AF163475	1.95	<0.0001
DKFZP434B2016	AL137655	1.94	0.0138
C7	NM_000587	1.74	0.0224
PDGFD	NM_025208	1.74	0.0070
LEPREL1	NM_018192	1.74	<0.0001
HSD17B1	NM_000413	1.74	<0.0001
ABCG2	NM_004827	1.68	0.0069
OR51B5	NM_001005567	1.67	0.0003
CFH	NM_000186	1.66	0.0372

**Table 3:** The most highly differentially expressed genes in the FGR samples

Gene name	Genbank	Fold-Change	*P *Value
**Up-regulated genes**			
CXCL9	NM_002416	2.44	0.0373
KRTAP26-1	NM_203405	1.91	0.0400
LOX	NM_002317	1.88	0.0020
CXCL13	NM_006419	1.74	0.0177
FPR3	NM_002030	1.73	0.0365
FLJ11827	XR_040865	1.71	0.0113
IFNG	NM_000619	1.70	0.0195
RGS1	NM_002922	1.69	0.0092
THBS1	NM_003246	1.90	0.0013
GZMK	NM_002104	1.67	0.0206
CYR61	NM_001554	1.65	0.0231
MSR1	NM_002445	1.63	0.0384
IFI44L	NM_006820	1.63	0.0347
TIMP1	NM_003254	1.62	0.0263
C1QB	NM_000491	1.60	0.0440
**Down-regulated genes**			
OR4F16	NM_001005277	1.85	0.0192
RPS6KA6	NM_014496	1.82	0.0029
PCDH11Y	NM_032971	1.69	0.0211
GABRE	U92285	1.64	0.0276
MGC35361	AK298753	1.64	0.0076
KIAA1467	NM_020853	1.63	0.0078
HIST1H2BK	NM_080593	1.61	0.0189
GABRA4	NM_000809	1.58	0.0018
LOC441233	AK128010	1.56	0.0321
PGAP1	NM_024989	1.55	0.0004
LRP2	NM_004525	1.55	0.0076
AGL	NM_000028	1.53	0.0069
CCDC125	NM_176816	1.53	0.0021
HYAL4	NM_012269	1.53	0.0102
C9orf131	AK090398	1.52	0.0034

**Table 4:** The most highly differentially expressed genes common to pre-eclampsia and FGR

Gene name	Genbank	Fold-Change (PE)	*P *Value (PE)	Fold-Change (FGR)	*P *Value (FGR)
**Up-regulated genes**					
LEP	NM_000230	10.94	<0.0001	3.56	0.0066
CGB	NM_000737	4.72	0.0011	2.50	0.0391
CGB5	NM_033043	4.71	0.0012	2.46	0.0436
CGB1	NM_033377	4.61	0.0010	2.38	0.0430
CGB7	NM_033142	4.43	0.0012	2.37	0.0445
CGB2	NM_033378	4.37	0.0007	2.37	0.0329
HTRA4	NM_153692	4.00	0.0002	1.93	0.0471
CRH	NM_000756	3.66	0.0008	2.36	0.0191
PAPPA2	NM_020318	2.55	0.0001	1.72	0.0125
NTRK2	NM_006180	2.50	<0.0001	1.58	0.0111
CP	NM_000096	2.44	0.0445	2.44	0.0446
FLT1	NM_002019	2.39	0.0001	1.51	0.0347
HTRA1	NM_002775	2.20	0.0015	1.67	0.0276
QPCT	NM_012413	2.12	0.0008	1.60	0.0240
ENG	NM_000118	2.03	0.0003	1.56	0.0143
**Down-regulated genes**					
ACOXL	NM_001105516	2.39	<0.0001	1.92	0.0003
GSTA3	NM_000847	2.09	0.0014	1.60	0.0318
HIST1H1T	NM_005323	2.06	0.0003	2.03	0.0004
FAM26D	NM_153036	2.05	0.0009	1.83	0.0040
SNORD116	AF241255	1.98	0.0203	2.07	0.0140
CATSPERB	NM_024764	1.98	0.0001	1.66	0.0023
WNT2	NM_003391	1.97	0.0023	1.58	0.0301
MUC15	NM_145650	1.89	0.0024	1.52	0.0371
SH3TC2	NM_024577	1.89	0.0012	1.63	0.0092
C12orf39	BC004336	1.85	0.0044	1.55	0.0366
TMEM136	NM_174926	1.84	<0.0001	1.61	0.0007
ZNF554	NM_001102651	1.76	0.0005	1.71	0.0009
APLN	NM_017413	1.76	0.0007	1.65	0.0020
NAALADL2	NM_207015	1.73	0.0039	1.71	0.0045
ZNF429	NM_001001415	1.70	0.0027	1.56	0.0096

**Figure 3:**
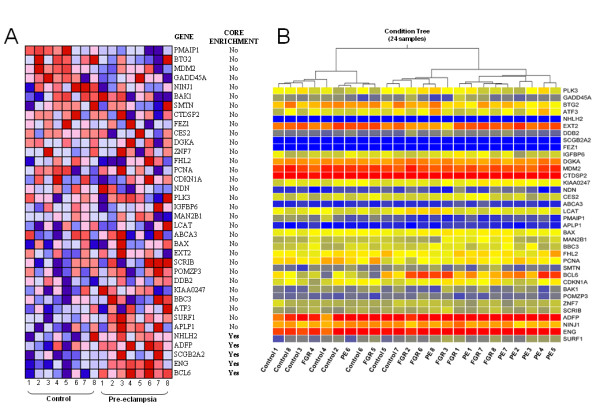
**GSEA for differentially expressed genes from pre-eclamptic placental tissue**. Red indicates up-regulation and blue indicates down-regulation. **A**. Heat map for the gene set, "Kannan_P53_up" which consisted of 36 genes (identified on the right). **B**. Hierarchical clustering of all subjects using the Kannan_P53_up gene set. Each column represents a single subject and each row a single gene. The height of the branches reflects the correlation coefficient.

To then investigate the differences between the biological processes underlying the development of pre-eclampsia and unexplained FGR, we first performed standard GO analyses, which did not reveal any specific biological processes or molecular functions for these disorders (Additional file 2, Tables S2 and Additional file 3, Tables S3). Next, we performed GSEA analysis to identify any gene sets that show significant differences for each sample group. The only significant functional gene set found for unexplained FGR was "Insulin_Adip_Insens_dn" (Table 5). This indicates that the genes downregulated by insulin play a prominent role in FGR and hence that the insulin pathway is specifically activated in unexplained cases of this disorder [27]. For the pre-eclampsia group, "Kannan_P53_up", consisting of *TP53*-responsive genes, was the only gene set found to be enriched (Table 6) [28]. Among the 36 genes in this set, five genes (*BCL6*, *ENG*, *SCGB2A2*, *ADFP *and *NHLH2*) were considered to contribute to the enrichment score (core enrichment), and the expression levels were significantly different in the genes *BCL6*, *ENG*, *ADFP*, *BAX *and *PMAIP1 *(Figure 3A). Clustering analysis using the total set of 36 genes assigned the pre-eclamptic samples and a subset of the unexplained FGR specimens to the same tree branches (Figure 3B).

**Table 5:** Gene sets enriched in FGR

Gene set	Nominal *P *value	FDR
**Up-regulated; 315/984**		
INSULIN_ADIP_INSENS_DN	<0.0001	0.237
LIZUKA_G1_SM_G2	<0.0001	0.272
AGEING_KIDNEY _DN	<0.0001	0.529
AGEING_KIDNEY_SPECIFIC_DN	<0.0001	0.643
CMV_HCMV_TIMECOURSE_24HRS_UP	<0.0001	0.705
BRACX_UP	<0.0001	0.814
UNDERHILL_PROLIFERAION	<0.0001	0.820
**Down-regulated; 669/984**		
IFNALPHA_RESIST_DN	<0.0001	1.000

Nominal *P *values <1% are listed.

**Table 6:** Gene sets enriched in pre-eclampsia

Gene set	Nominal *P *value	FDR
**Up-regulated; 315/984**		
KANNAN_P53_UP	<0.0001	0.235
**Down-regulated; 669/984**		
LIZUKA_G1_SM_G2	0.009	1.000
DORSAM_HOXA9_UP	0.009	1.000

Nominal *P *values <1% are listed.

**Figure 4:**
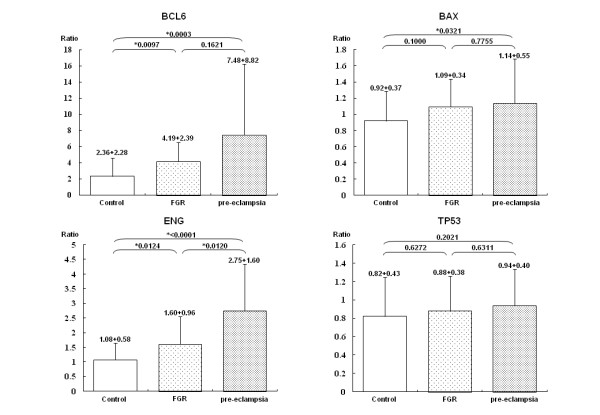
**qRT-PCR analysis of *TP53*-related genes**. These data were compared for normotensive control pregnancies (open bars; *n *= 45), FGR (dotted bars; *n *= 16) and pre-eclampsia (grey bars; *n *= 41). Each bar represents the mean value and each vertical line indicates the standard deviation. *P < 0.05, ANOVA test. The up-regulation of ADFP was described also in our previous study [16].

To confirm the activation of the *TP53 *pathway in pre-eclampsia, qRT-PCR for *TP53*-related genes was performed using an increased number of samples (41 pre-eclampsia, 16 FGR and 45 normotensive pregnancy specimens) to validate our microarray results. The expression of *TP53 *itself did not change among the three groups (Figure 4 and Additional file 4, Figure S1). However, a significant up-regulation of the downstream genes *BCL6*, *ENG*, *ADFP, APLP1, SURF1 *and *BAX *was confirmed in pre-eclampsia. Among these genes, the apoptosis-related genes *BCL6*, *ENG *and *BAX *appeared to be upregulated also in unexplained FGR, but at lower levels compared with the pre-eclamptic placentas. Among the other genes related to apoptosis, *FASLG *and *P53AIP1 *were also found to be up-regulated in pre-eclampsia (Table 7). However, the genes encoding caspases were not up-regulated, suggesting that apoptosis itself is not executed in pre-eclampsia.

**Table 7:** Microarray analysis of apoptosis-associated genes

	Control (n = 8)	PE (n = 8)	*P *value
TP53 (NM_000546)	8.04 ± 0.10	7.93 ± 0.25	n.s.
MDM2 (NM_002392)	8.96 ± 0.15	8.79 ± 0.20	n.s.
FAS (NM_000043)	6.44 ± 0.19	6.29 ± 0.38	n.s.
FASLG (NM_000639)	5.25 ± 0.09	5.50 ± 0.26	0.030
BCL2 (NM_000633)	8.42 ± 0.16	8.38 ± 0.3	n.s.
MCL1 (NM_021960)	10.07 ± 0.16	10.05 ± 0.27	n.s.
BID (NM_197966)	6.21 ± 0.06	6.21 ± 0.15	n.s.
BAD (NM_004322)	7.16 ± 0.10	7.35 ± 0.25	n.s.
BAK1 (NM_001188)	6.59 ± 0.20	6.51 ± 0.26	n.s.
CASP2 (NM_032982)	7.81 ± 0.07	7.72 ± 0.19	n.s.
CASP3 (NM_004346)	7.22 ± 0.21	7.11 ± 0.25	n.s.
CASP6 (NM_001226)	4.71 ± 0.3	4.77 ± 0.36	n.s.
CASP7 (NM_033338)	7.18 ± 0.10	7.17 ± 0.25	n.s.
CASP8 (NM_001228)	6.75 ± 0.14	6.66 ± 0.22	n.s.
CASP9 (NM_001229)	7.18 ± 0.17	7.18 ± 0.19	n.s.
CASP10 (NM_032977)	5.58 ± 0.14	5.47 ± 0.26	n.s.
XIAP (NM_001167)	7.09 ± 0.23	7.08 ± 0.32	n.s.
BIRC5 (NM_001168)	5.97 ± 0.23	6.07 ± 0.29	n.s.
APAF1 (NM_181861)	6.85 ± 0.11	6.82 ± 0.36	n.s.
P53AIP1 (NM_022112)	4.16 ± 0.14	4.42 ± 0.29	0.025

Data for normalized signal intensities are shown.

## Discussion

To date, a large body of evidence has accumulated with regards to the global expression profiles of the pre-eclamptic placenta, and several important genes have been identified via this strategy [16,29,30]. On the other hand, although there are a comparable number of reports showing microarray analysis for the FGR placenta, these investigations have not been as effective in identifying potentially causative genes for this disease. Previous studies have documented that genes involved in hypoxia or metabolism-related genes are common to the group of differentially expressed genes in the FGR placenta [31-34]. However, these findings may require careful interpretation, since the expression of these genes is often affected by sampling site bias [35,36]. In addition, although the question of whether pre-eclampsia and FGR share a common etiology is not a long-standing issue, there have been few comparative expression profiles undertaken for these disorders, and the results are not consistent [37,38].

In our current study, comparative analysis of the global expression profiles of pre-eclampsia and FGR indicate a common pathophysiology. This is based on our findings that 1) pre-eclampsia and a subset of FGR samples show a high correlation coefficient for their overall microarray data and are clustered into the same category, and 2) most of the genes known to be up-regulated in pre-eclampsia were found to be up-regulated in FGR. A possible interpretation might be that pre-eclampsia and a subset of FGR are in fact the same disorder with the same etiology, and that the apparent differences depend on the degree of severity. Alternatively, it is possible that these two disorders are related conditions with the same etiology and differing only by the presence of maternal hypertension.

It was important to then address whether the presence or absence of maternal symptoms determines the phenotypic difference between pre-eclampsia and unexplained FGR. Although standard GO analyses did not reveal specific biological processes or molecular functions, the data from GSEA analyses show that *TP53 *downstream genes are more highly activated in pre-eclampsia than in FGR. As *TP53 *itself is not up-regulated at the mRNA level, this activation is likely due to stabilization of the p53 protein, which has a known short half-life [39]. Up-regulated genes in pre-eclampsia also included *BAX *and *BCL6*, which are known to regulate apoptosis [40,41]. A prevailing hypothesis is that trophoblast hypoxia due to abnormal spiral arteries triggers apoptosis leading to the onset of pre-eclampsia [42]. The TP53 pathway may contribute to this process. The current literature does not support the contribution of *TP53 *to pre-eclampsia but a considerable body of evidence indicates that the apoptosis of trophoblasts itself is increased in the pre-eclamptic placenta [43-45].

Our present data indicate that most of the genes known to be up-regulated in the placenta in pre-eclampsia are also up-regulated in unexplained FGR placentas. These genes include *FLT1 *and *ENG*, which encode soluble proteins that are detected in the maternal circulation and serve as anti-angiogenic factors [6,7]. These data are consistent with previously published findings [46-49]. Although conflicting data have also been reported [50,51], it might be possible that the FGR samples in these reports might be derived from late-onset cases that are fundamentally different at the molecular level [52]. High levels of these circulating factors are likely to induce maternal systemic endothelial dysfunction. There is little doubt also that the apoptosis of trophoblasts is associated with cell turnover in the placental villi [53]. This apoptotic process halts at an early stage, and dissolution and fusion of the plasma membrane leads to generation of syncytiotrophoblasts, with accompanying microparticles shedding to the maternal circulation [54,55]. Excess shedding, possibly due to a hypoxia-induced increased turnover of syncytiotrophoblasts, is often observed in the pre-eclamptic placenta and manifests as syncytial knots [56]. It is possible therefore that an up-regulation of the *TP53 *pathway induces the deportation of microparticles, including high levels of anti-angiogenic factors, and that this might facilitate maternal systemic symptoms in pre-eclampsia [57,58].

Several reports have now indicated that placentas from FGR pregnancies without pre-eclampsia also manifest increased apoptosis or the up-regulation of *TP53 *[59-61]. However, the levels of the apoptotic response in FGR appear to be less severe than in pre-eclampsia [62]. In this context, our present data suggest that a subset of unexplained FGR might possibly be pre-eclampsia with mild maternal symptoms that do not fit the currently accepted disease criteria. Our qRT-PCR data showing that *TP53*-downstream genes are highly up-regulated in pre-eclampsia and in some cases of unexplained FGR lend moderate support to this hypothesis. Hence, in spite of the preliminary nature of the study due to the small sample size, these data warrant further investigation.

## Competing interests

The authors declare that they have no competing interests.

## Authors' contributions

HN conceived the project, participated in its design and coordination, and analyzed the data. SO, MS and TK carried out the overall molecular experiments including the microarray analysis. TS carried out the operations, and participated in the design and coordination of the study. HK conceived the project, participated in its design and coordination, and helped to draft the manuscript. YU participated in the design and coordination of the project and helped to draft the manuscript. All of the authors read and approved the final manuscript.

## Supplementary Material

Additional file 1 **Table S1: List of differentially expressed genes common to pre-eclampsia and FGR**. Click here for file

Additional file 2 **Table S2: List of biological processes by gene ontology**. Click here for file

Additional file 3 **Table S3: List of molecular functions by gene ontology**. Click here for file

Additional file 4 **Figure S1: qRT-PCR analysis of *TP53*-related genes**. Click here for file
