# Supplementary material for: Comparative gene expression profiling of placentas from patients with severe pre-eclampsia and unexplained fetal growth restriction
Source: Reprod Biol Endocrinol. 2011 Aug 2;9:107. doi: 10.1186/1477-7827-9-107 (PMC3199758; doi:10.1186/1477-7827-9-107)
Supplement: Additional file 1 — Table S1: List of differentially expressed genes common to pre-eclampsia and FGR. [file 1477-7827-9-107-S1.DOC]

**Supplemental table 1. List of differentially expressed genes common to pre-eclampsia and FGR.**

**Up-regulated genes**

| **Gene name** | **RefSeq** | **Fold-Change**  **(PE)** | ***P* Value**  **(PE)** | **Fold-Change**  **(FGR)** | ***P* Value**  **(FGR)** |
| --- | --- | --- | --- | --- | --- |
| LEP | NM_000230 | 10.94 | <0.0001 | 3.56 | 0.0066 |
| CGB | NM_000737 | 4.72 | 0.0011 | 2.50 | 0.0391 |
| CGB5 | NM_033043 | 4.71 | 0.0012 | 2.46 | 0.0436 |
| CGB1 | NM_033377 | 4.61 | 0.0010 | 2.38 | 0.0430 |
| CGB7 | NM_033142 | 4.43 | 0.0012 | 2.37 | 0.0445 |
| CGB2 | NM_033378 | 4.37 | 0.0007 | 2.37 | 0.0329 |
| HTRA4 | NM_153692 | 4.00 | 0.0002 | 1.93 | 0.0471 |
| CRH | NM_000756 | 3.66 | 0.0008 | 2.36 | 0.0191 |
| PAPPA2 | NM_020318 | 2.55 | 0.0001 | 1.72 | 0.0125 |
| NTRK2 | NM_006180 | 2.50 | <0.0001 | 1.58 | 0.0111 |
| CP | NM_000096 | 2.44 | 0.0445 | 2.44 | 0.0446 |
| FLT1 | NM_002019 | 2.39 | 0.0001 | 1.51 | 0.0347 |
| HTRA1 | NM_002775 | 2.20 | 0.0015 | 1.67 | 0.0276 |
| QPCT | NM_012413 | 2.12 | 0.0008 | 1.60 | 0.0240 |
| Unknown gene on 2p11.2 | ENST00000421040 | 2.08 | 0.0023 | 1.60 | 0.0395 |
| ENG | NM_000118 | 2.03 | 0.0003 | 1.56 | 0.0143 |
| NDRG1 | NM_006096 | 2.02 | 0.0001 | 1.55 | 0.0105 |
| BHLHB2 | NM_003670 | 1.95 | 0.0004 | 1.58 | 0.0100 |
| AQP1 | NM_198098 | 1.84 | 0.0007 | 1.86 | 0.0006 |
| Unknown gene on 9q22.1 |  | 1.77 | 0.0206 | 2.16 | 0.0028 |
| Unknown gene on 2p11.2 | ENST00000390264 | 1.68 | 0.0041 | 1.71 | 0.0031 |
| Unknown gene on 15q14 |  | 1.63 | 0.0098 | 1.52 | 0.0230 |
| ST8SIA6 | NM_001004470 | 1.63 | 0.0012 | 1.56 | 0.0027 |
| DUSP1 | NM_004417 | 1.61 | 0.0066 | 1.55 | 0.0116 |
| Unknown gene on 1p22.1 | ENST00000384649 | 1.53 | 0.0382 | 1.76 | 0.0075 |
| Unknown gene on 12q24.11 |  | 1.53 | 0.0032 | 1.64 | 0.0009 |
| C5orf23 | BC022250 | 1.53 | 0.0432 | 1.57 | 0.0318 |
| Unknown gene on 9q22.31 |  | 1.51 | 0.0081 | 1.71 | 0.0009 |
| Unknown gene on 10q24.32 | ENST00000410482 | 1.50 | 0.0107 | 1.55 | 0.0069 |

**Down-regulated genes**

| **Gene name** | **RefSeq** | **Fold-Change**  **(PE)** | ***P* Value**  **(PE)** | **Fold-Change**  **(FGR)** | ***P* Value**  **(FGR)** |
| --- | --- | --- | --- | --- | --- |
| Unknown gene on 7p22.1 | ENST00000365403 | 2.44 | 0.0006 | 1.86 | 0.0116 |
| Unknown gene on 5q32 |  | 2.43 | 0.0051 | 2.06 | 0.0196 |
| ACOXL | NM_001105516 | 2.39 | <0.0001 | 1.92 | 0.0003 |
| GSTA3 | NM_000847 | 2.09 | 0.0014 | 1.60 | 0.0318 |
| HIST1H1T | NM_005323 | 2.06 | 0.0003 | 2.03 | 0.0004 |
| FAM26D | NM_153036 | 2.05 | 0.0009 | 1.83 | 0.0040 |
| SNORD116 | AF241255 | 1.98 | 0.0203 | 2.07 | 0.0140 |
| CATSPERB | NM_024764 | 1.98 | 0.0001 | 1.66 | 0.0023 |
| WNT2 | NM_003391 | 1.97 | 0.0023 | 1.58 | 0.0301 |
| Unknown gene on 3q13.12 |  | 1.91 | 0.0202 | 1.72 | 0.0485 |
| MUC15 | NM_145650 | 1.89 | 0.0024 | 1.52 | 0.0371 |
| SH3TC2 | NM_024577 | 1.89 | 0.0012 | 1.63 | 0.0092 |
| C12orf39 | BC004336 | 1.85 | 0.0044 | 1.55 | 0.0366 |
| TMEM136 | NM_174926 | 1.84 | <0.0001 | 1.61 | 0.0007 |
| ZNF554 | NM_001102651 | 1.76 | 0.0005 | 1.71 | 0.0009 |
| APLN | NM_017413 | 1.76 | 0.0007 | 1.65 | 0.0020 |
| NAALADL2 | NM_207015 | 1.73 | 0.0039 | 1.71 | 0.0045 |
| Unknown gene on 16q22.1 | AF370400 | 1.71 | 0.0122 | 1.65 | 0..0180 |
| ZNF429 | NM_001001415 | 1.70 | 0.0027 | 1.56 | 0.0096 |
| Unknown gene on 16p13.13 | AF258574 | 1.69 | 0.0182 | 1.54 | 0.0471 |
| Unknown gene on 7p22.1 | ENST00000458975 | 1.68 | 0.0275 | 1.66 | 0.0311 |
| F5 | NM_000130 | 1.66 | 0.0023 | 1.79 | 0.0006 |
| TMEM168 | NM_022484 | 1.64 | 0.0006 | 1.59 | 0.0011 |
| FLJ13744 | BC070061 | 1.63 | 0.0163 | 1.65 | 0.0145 |
| RASSF6 | NM_201431 | 1.59 | 0.0023 | 1.62 | 0.0015 |
| PCDH11X | NM_032967 | 1.56 | 0.0355 | 1.74 | 0.0105 |
| LGR5 | NM_003667 | 1.56 | 0.0085 | 1.53 | 0.0108 |
| KIAA0746 | NM_015187 | 1.53 | 0.0119 | 1.53 | 0.0118 |
| KCNK17 | NM_031460 | 1.52 | 0.0204 | 2.01 | 0.0004 |
| DSC3 | NM_024423 | 1.51 | 0.0256 | 1.56 | 0.0165 |
| Unknown gene on 14q22.1 |  | 1.51 | 0.0217 | 1.80 | 0.0018 |
| ACPP | NM_001099 | 1.50 | 0.0010 | 1.64 | 0.0001 |
