# Supplementary material for: Comparative gene expression profiling of placentas from patients with severe pre-eclampsia and unexplained fetal growth restriction
Source: Reprod Biol Endocrinol. 2011 Aug 2;9:107. doi: 10.1186/1477-7827-9-107 (PMC3199758; doi:10.1186/1477-7827-9-107)
Supplement: Additional file 2 — Table S2: List of biological processes by gene ontology. [file 1477-7827-9-107-S2.DOC]

**Supplemental table 2. List of biological processes by gene ontology.**

| **Biological process (GO ID)** | **Enrichment score** | **Enrichment p-value** | **Genes in list** |
| --- | --- | --- | --- |
| Regulation of cell growth (ID:1558) | 8.074 | 0.0003 | PAPPA2, HTRA1, HTRA4 |
| Patterning of blood vessels (ID: 1569) | 7.037 | 0.0009 | FLT1, ENG |
| Potassium ion transmembrane transport (ID: 71805) | 6.588 | 0.0014 | KCNK17, AQP1 |
| Cellular response to retinoic acid (ID: 71300) | 6.588 | 0.0014 | AQP1, WNT2 |
| Positive regulation of fibroblast proliferation (ID: 48146) | 6.033 | 0.0024 | AQP1, WNT2 |
| Negative regulation of transforming growth factor beta receptor signaling pathway (ID: 30512) | 5.974 | 0.0025 | HTRA1, ENG |
| Proteolysis (ID: 6508) | 5.824 | 0.0030 | PAPPA2, HTRA1, QPCT, NAALADL2, HTRA4 |
| Cell differentiation (ID: 30154) | 5.469 | 0.0042 | PAPPA2, FLT1, CATSPERB, HIST1H1T, NTRK2 |
| Glycolipid biosynthetic process (ID: 9247) | 5.021 | 0.0066 | ST8SIA6 |
| Intracellular receptor mediated signaling pathway (ID: 30522) | 5.021 | 0.0066 | FLT1 |
