# Supplementary material for: Comparative gene expression profiling of placentas from patients with severe pre-eclampsia and unexplained fetal growth restriction
Source: Reprod Biol Endocrinol. 2011 Aug 2;9:107. doi: 10.1186/1477-7827-9-107 (PMC3199758; doi:10.1186/1477-7827-9-107)
Supplement: Additional file 3 — Table S3: List of molecular functions by gene ontology. [file 1477-7827-9-107-S3.DOC]

**Supplemental table 3. List of molecular functions by gene ontology.**

| **Biological process (GO ID)** | **Enrichment score** | **Enrichment p-value** | **Genes in list** |
| --- | --- | --- | --- |
| Hormone activity (ID: 5179) | 17.185 | <0.0001 | CGB2, CGB1, CGB7, LEP, CRH, APLN |
| Insulin-like growth factor binding (ID: 5520) | 6.939 | 0.0010 | HTRA1, HTRA4 |
| Potassium channel activity (ID: 5267) | 6.033 | 0.0024 | KCNK17, AQP1 |
| Transmembrane receptor protein tyrosine kinase activity (ID: 4714) | 5.861 | 0.0029 | FLT1, NTRK2 |
| Peptidase activity (ID: 8233) | 5.358 | 0.0047 | PAPPA2, HTRA1, QPCT, NAALADL2, HTRA4 |
| Potassium ion transmembrane transporter activity (ID: 15079) | 5.021 | 0.0066 | AQP1 |
| Neurotrophin binding (ID: 43121) | 5.021 | 0.0066 | NTRK2 |
| Neurotrophin receptor activity (ID: 5030) | 5.021 | 0.0066 | NTRK2 |
| Copper ion binding (ID: 5507) | 4.961 | 0.0070 | F5, CP |
| Protein-hormone receptor activity (ID: 16500) | 4.734 | 0.0088 | LGR5 |
