# Supplementary material for: Comparative gene expression profiling of placentas from patients with severe pre-eclampsia and unexplained fetal growth restriction
Source: Reprod Biol Endocrinol. 2011 Aug 2;9:107. doi: 10.1186/1477-7827-9-107 (PMC3199758; doi:10.1186/1477-7827-9-107)
Supplement: Additional file 4 — Figure S1: qRT-PCR analysis of TP53-related genes. [file 1477-7827-9-107-S4.DOC]

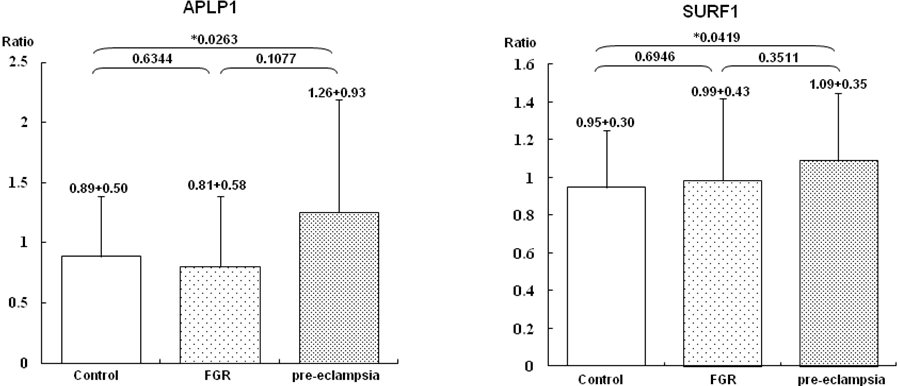


**Supplemental figure 1.** qRT-PCR analysis of *TP53*-related genes. These data were compared for normotensive control pregnancies (open bars; *n* = 45), FGR (dotted bars; *n* = 16) and pre-eclampsia (grey bars; *n* = 41). Each bar represents the mean value and each vertical line indicates the standard deviation. **P*<0.05, ANOVA test.
